# Prenatal enrichment and recovery from perinatal cortical damage: effects of maternal complex housing

**DOI:** 10.3389/fnbeh.2014.00223

**Published:** 2014-06-24

**Authors:** Robbin L. Gibb, Claudia L. R. Gonzalez, Bryan Kolb

**Affiliations:** Department of Neuroscience, Canadian Centre for Behavioural Neuroscience, University of LethbridgeLethbridge, AB, Canada

**Keywords:** complex housing, cortical injury, Golgi, recovery, plasticity, prenatal

## Abstract

Birth is a particularly vulnerable time for acquiring brain injury. Unfortunately, very few treatments are available for those affected. Here we explore the effectiveness of *prenatal* intervention in an animal model of early brain damage. We used a complex housing paradigm as a form of prenatal enrichment. Six nulliparous dams and one male rat were placed in complex housing (condomom group) for 12 h per day until the dams' delivered their pups. At parturition the dams were left in their home (standard) cages with their pups. Four dams were housed in standard cages (cagemom group) throughout pregnancy and with their pups until weaning. At postnatal day 3 (P3) infants of both groups received frontal cortex removals or sham surgery. Behavioral testing began on P60 and included the Morris water task and a skilled reaching task. Brains were processed for Golgi analyses. Complex housing of the mother had a significant effect on the behavior of their pups. Control animals from the condomom group outperformed those of the cagemom group in the water task. Condomom animals with lesions performed better than their cagemom cohorts in both the water task and in skilled reaching. Condomom animals showed an increase in cortical thickness at anterior planes and thalamic area at both anterior and posterior regions. Golgi analyses revealed an increase in spine density. These results suggest that prenatal enrichment alters brain organization in manner that is prophylactic for perinatal brain injury. This result could have significant implications for the prenatal management of infants expected to be at risk for difficult birth.

## Introduction

Environmental effects on behavior and cerebral architecture in control (uninjured) animals have been well documented (Hebb, 1949; Rosenzweig et al., 1962; Rosenzweig, 1971; Greenough and Volkmar, 1973; Sale et al., 2009, 2014, for a review Sale et al., 2009, 2014). These studies demonstrated that complex housing improved behavioral performance and increased such anatomical features as brain weight, cortical thickness, acetylcholinesterase density, and number of synapses. Experiments designed to assess the effect of complex housing on functional recovery after brain damage in adulthood have shown modest, yet, significant improvements (e.g., Kolb and Gibb, 1991; Will and Kelche, 1992; Johansson and Belichenko, 2002; Fares et al., 2013). Environmental treatments for brain-damaged pups, however, have shown greater success in ameliorating functional deficits (Comeau et al., 2008; Saucier et al., 2010; Rojas et al., 2013). For example, complex housing of weanling rats has been shown to reverse some of the behavioral deficits caused by early cortical lesion (Comeau et al., 2008). Furthermore, environmental enrichment has been shown to reverse some of the effects of prenatal stress on a variety of cognitive, motor, and emotional tests (Chapillon et al., 2002; Morley-Fletcher et al., 2003; Fox et al., 2006; Li et al., 2012).

Although we are unaware of studies examining the effect of prenatal enrichment on recovery from cerebral injury, a study by Kiyono et al. (1985) demonstrated that offspring born to complex housed females were advantaged compared to offspring born to standard-housed dams when tested in adulthood in a Hebb-Williams maze. The effect of prenatal enrichment on learning was also examined by Koo et al. (2003). This study reported that spatial learning and memory, and the expression of neuronal cell adhesion molecules (used as a marker of synaptic plasticity) were enhanced. A recent study investigated how complex housing during gestation modulated the effects of prenatal stress in offspring rats (Li et al., 2012). Results showed partial improvement in anxiety scores and cognitive performance in the Morris water task. The behavioral improvements were accompanied by reversal of dendritic spine loss in the CA1 and dentate gyrus of the hippocampus. These experiments led us to hypothesize that prenatal complex housing could provide some measure of protection against later perinatal brain injury.

**Figure 1:**
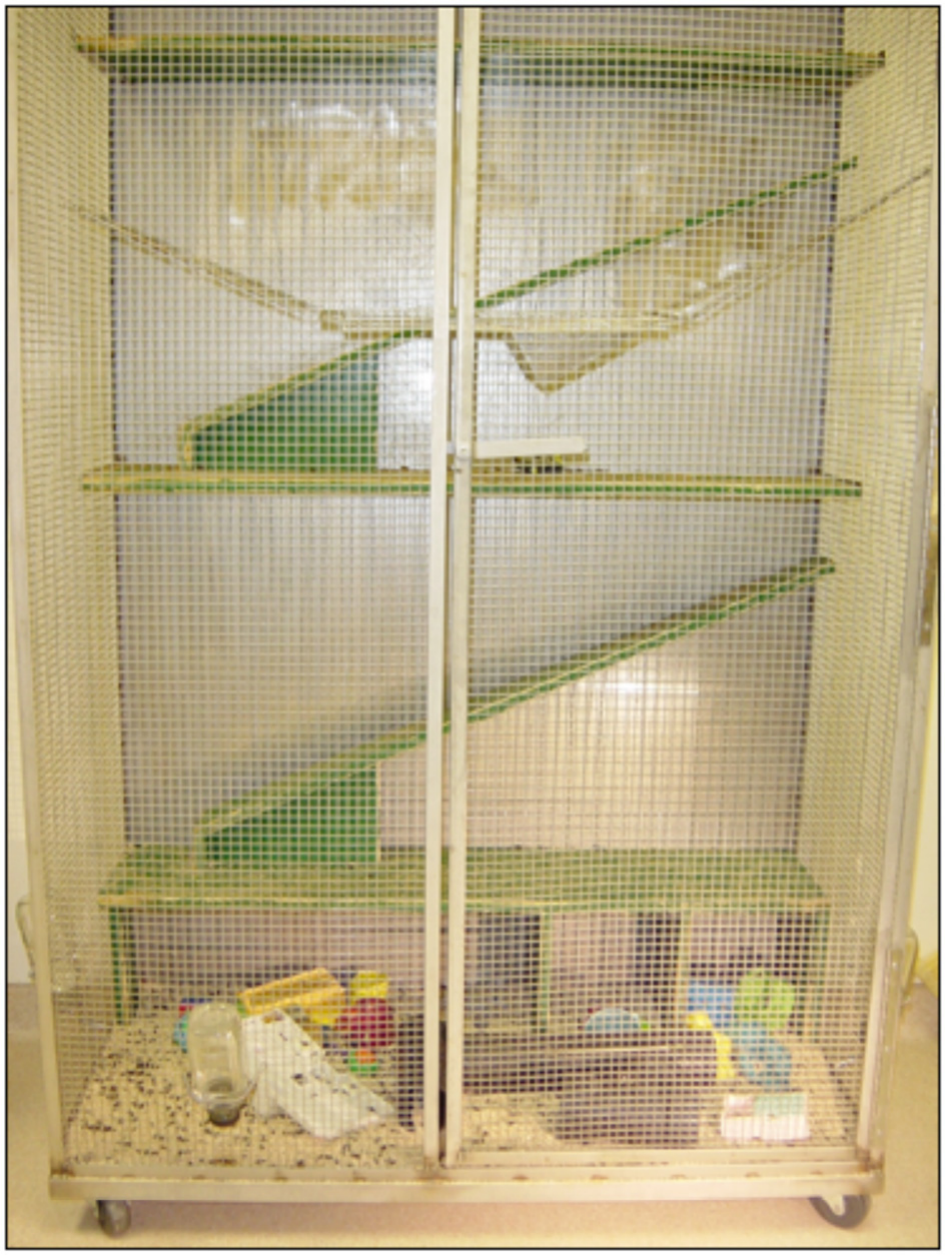
**Complex housing for rats (Condominiums)**. The hardware cloth construction and a series of runways and swings allowed exploration of the condominiums in all dimensions. PVC pipes provided dark shelter for inhabitants. A variety of hard plastic toys were placed on the condo floor and were changes weekly to provide additional stimulation.

Animals with frontal cortex lesions at P3 show abysmal functional recovery on cognitive, motor, and species typical behaviors as well as abnormal cortical morphology (e.g., Kolb and Gibb, 1990, 2010; Kolb, 1995; Gibb and Kolb, 2005). In the current study we used this model of perinatal brain injury to investigate the possibility that prenatal enrichment could improve behavioral recovery in adulthood. To this end, female rats were introduced to complex housing (see Figure 1) for a week prior to and throughout the duration of their pregnancy. Half of the pups born to mothers housed in the complex environment or standard caging received removals of frontal cortex at P3. On P60, animals were tested in a well-known task of cognitive function (Morris, 1981) followed by the assessment of motor performance on a forepaw reaching task (Whishaw et al., 1986). At the conclusion of behavioral testing, animals were euthanized and their body and brain weight were recorded. Brains were processed for using a Golgi method (Gibb and Kolb, 1998) for spine analysis. Lesion size, cortical thickness, and thalamic measures were obtained from stained tissue.

## Materials and methods

### Subjects and housing procedures

Six adult female Long-Evans rats (90 days old) were placed in complex housing for 1 week prior to the introduction of an adult male rat. All experiments were carried out in accordance with the Canadian Council of Animal Care and approved by the University of Lethbridge Animal Care Committee. The complex housing enclosure (condominium) was a large pen measuring 63 × 148 × 187 cm. Three walls were constructed of sturdy wire mesh and the fourth wall consisted of plywood overlaid with blue arborite®. Within these enclosures were swings, plastic pipes, ramps, and runways, and a variety of “toys” that were moved and/or changed weekly when the pen was cleaned (Figure 1). The females remained in the complex housing for 24 h per day (12 h light/12 h dark) for the first 10 days (7 days prior to mating and 3 days with the sire) and then were removed from the condominiums and placed in standard plastic breeding cages for 12 h each day during the dark cycle as described in Mychasiuk et al. (2012). This procedure was introduced to acclimatize the mothers to the cages they would be occupying once their pups were born. They were returned to the condominiums each morning and this pattern of housing changes continued up to the day of parturition. It was noted that the pregnant dams used all levels of the condominium right up to parturition. The male remained in the condominium until all dams were delivered of their pups. Food treats (Froot Loops®) were given to the dams each time they were moved to the standard cages (the control dams also received the same food reward in their cages at the same time). Two females delivered on the same day, 1 delivered 9 days later and two more 14 days after the first pups were born. One female did not become pregnant. In total 42 pups were born to the 5 females and within these litters there were 24 female and 18 male pups (condomom animals). Within the female group, 14 received postnatal day 3 (P3) frontal removals and 10 were sham-operates. In the male group, 9 received the frontal surgery and 9 were sham-operates. The dams remained with their pups until postnatal day 21 (P21) when the pups were weaned and placed in standard plastic cages (3–6 animals per cage). No further enrichment was experienced by the offspring animals and they were subsequently handled only when their cages were cleaned. Four litters of animals born to mothers in standard cages (cagemom animals) served as the control group for this experiment. There were a total of 23 animals in these litters (11 female, 12 male). Within the female group there were six frontal operates and five shams and in the male group, seven frontals and five shams.

### Surgery

As in previous studies (Gibb and Kolb, 2005; Kolb and Gibb, 2010) on postnatal day 3 (P3) the pups were removed from the nest and cooled in a Thermatron® cooling chamber until their core temperature reached approximately 20°C. The lesion animals had their scalp opened then the frontal bone carefully removed after it was incised with iris scissors. The medial frontal cortex was then removed bilaterally with gentle aspiration. The tissue targeted for removal was the medial subfield of the prefrontal cortex including (Zilles, 1985) regions Cg1, Cg3, and PL as well as the medial portion of Fr2 of the motor cortex. After aspiration of the cortical tissue, the animals' scalp was sutured with silk thread (00000) drawn by a very fine needle. The remaining control animals underwent a sham surgical procedure in which the scalp was opened and then sutured closed but the skull was not removed. These animals were identified by removal of the tip of the outer toe on their right rear foot.

### Behavioral methods

#### Morris water task

Beginning at P60 animals were trained on the Morris water task using a similar procedure to that described by Sutherland et al. (1983) based on the original task described by Morris (1981). The maze consisted of a circular pool (1.5 m diameter × 0.5 m deep) with smooth white walls. The pool was filled with approximately 25°C water mixed with 500 ml of skim milk powder, used to render the water opaque. A clear Plexiglas® platform (11 × 12 cm) was placed in a constant position inside the pool approximately 30 cm from the pool wall. The water level was adjusted so that the platform was invisible to a viewer outside the pool and to a rat swimming in the water. A trial consisted of placing a rat into the water facing the pool edge at one of four compass locations (north, south, east, or west) around the pool's perimeter. Within a block of four trials each rat started at the four locations in random sequence, and each rat was tested for four trials a day over five consecutive days. If on a particular trial a rat found the platform, it was permitted to remain on it for 10 s. A trial was terminated if the rat failed to find the platform after 90 s. Each rat was returned to its holding cage for approximately 5 min before the next trial commenced. The swim path for each rat on every trial was recorded using a Poly Track video tracking system (San Diego Instruments) which tracks the swim path and records the latency, distance, and dwell time within each quadrant.

#### Whishaw tray reaching

Following water maze training, animals were trained in a skilled reaching task developed by Whishaw et al. (1991). In this task rats were trained to retrieve chicken feed through metal bars at the front of the Plexiglas® training cage (28 cm deep × 20 cm wide × 25 cm high). The front of each cage was constructed with 2 mm bars separated from each other by 1 cm, edge to edge and the floor was constructed of wire mesh. A tray (5 cm deep × 2 cm wide × 1 cm high) containing chicken feed pellets was mounted in the front of each cage. To obtain food, the rats had to extend their forelimbs through the bars, grasp, and retract the food pellet. The food tray was mounted on runners to adjust the distance of the food from the bars. Distance adjustment ensured that each rat could not simply rake the food into the cage. Any pellets that the rat dropped inside the cage were irretrievably lost through the mesh on the floor and the animal would have to reach again. During the first few days the rats were pre-trained in pairs in the reaching cages for a period of one half hour per day. Once reach training commenced, the animals were provided with 15 grams of rat chow daily following the training period. The rats were subsequently trained individually for one half hour per day and then at the end of a 2-week training period their performance was videotaped for a 5-min interval. Each time the rat reached through the bars whether or not food was obtained was scored as a “reach,” and each time food was successfully returned to the cage and consumed was scored as a “hit.” The percentage of hits to total reaches was then calculated for each animal's taped performance.

### Anatomical methods

#### Histological procedures

At the conclusion of behavioral testing animals were given an overdose of sodium pentobarbital and intracardially perfused with a solution of 0.9% saline. The trimmed brains were weighed and then immersed whole in 20 mls of Golgi-Cox solution. The brains were then stored (in the dark) in the Golgi-Cox fixative for 14 days before being transferred to a solution of 30% sucrose for 7 days. The tissue was cut at 200 μm on a Vibratome™ then developed using a method described by Gibb and Kolb (1998).

### Anatomical analyses

#### Cortical thickness measurements

Cortical thickness measurements were obtained from the Golgi-Cox stained coronal sections projected on a Zeiss-Jena MF2 projector at a magnification of 20× (following the method described by Stewart and Kolb (1988). Briefly, three cortical measures were made at points medial, central, and lateral on five sections of tissue identified by the following landmarks; Plane 1: first caudate-putamen visible, Plane 2: anterior commissure, Plane 3: first hippocampal section, Plane 4: posterior commissure, Plane 5: last hippocampal section (see **Figure 4**). A plastic metric ruler was used to measure from the edge of the cortex to the edge of the white matter. An average for each plane and for each animal was calculated and used for statistical comparison.

#### Assessment of lesion size

Lesion size was estimated for all animals in lesion groups from whole brain pictures taken with a digital camera. The digital images were opened in the Scion Image program then the lesion traced around its perimeter and the area analyzed. The same procedure was repeated for the whole brain (including the lesion area but excluding the cerebellum) and then the ratio of lesion/brain was calculated for an estimation of lesion size.

#### Assessment of thalamic size

Thalamic cross-sectional area was measured from two coronal sections stained with Golgi-Cox using a Kodak digital camera to capture the image and the Scion Image program to measure thalamic area. One measure was taken of the anterior thalamus (approximately −1.80 mm from the Bregma). At this level the thalamus was defined using the internal capsule as a boundary to derive the area of the thalamus. The second measure was made in posterior thalamus at approximately −4.30 mm from the Bregma (as described in a study by Kolb and Whishaw (1981). At this level, the medial lemniscus provided the boundary from which to derive thalamic area.

#### Morphological analyses of neurons

As described in Gibb and Kolb (2005), spine density was measured from one apical dendritic branch in the terminal tuft and one basilar terminal branch of Par1 of the parietal cortex (Zilles, 1985). For each animal, five apical and five basilar branches were drawn from each hemisphere (10 apical and 10 basilar in total). Spine density measures were made from a segment greater than 10 μm in length, and usually about 50 μm. The dendrite was traced (1000×) using a camera lucida and the exact length of the dendritic segment calculated by placing a thread along the drawing and then measuring the thread length. Spine density was expressed as the number of spines per 10 μm. No attempt was made to correct for spines hidden beneath or above the dendritic segment so the spine density values are likely to underestimate the actual density of the dendritic spines.

### Statistical analyses

All statistical analyses were ANOVA's performed on Statsview 5®. If an ANOVA did not show a significant effect of sex, the data were collapsed across this variable to increase the number of subjects per group and to simplify the analysis.

## Results

### Behavioral results

#### Morris water task

**Figure 2:**
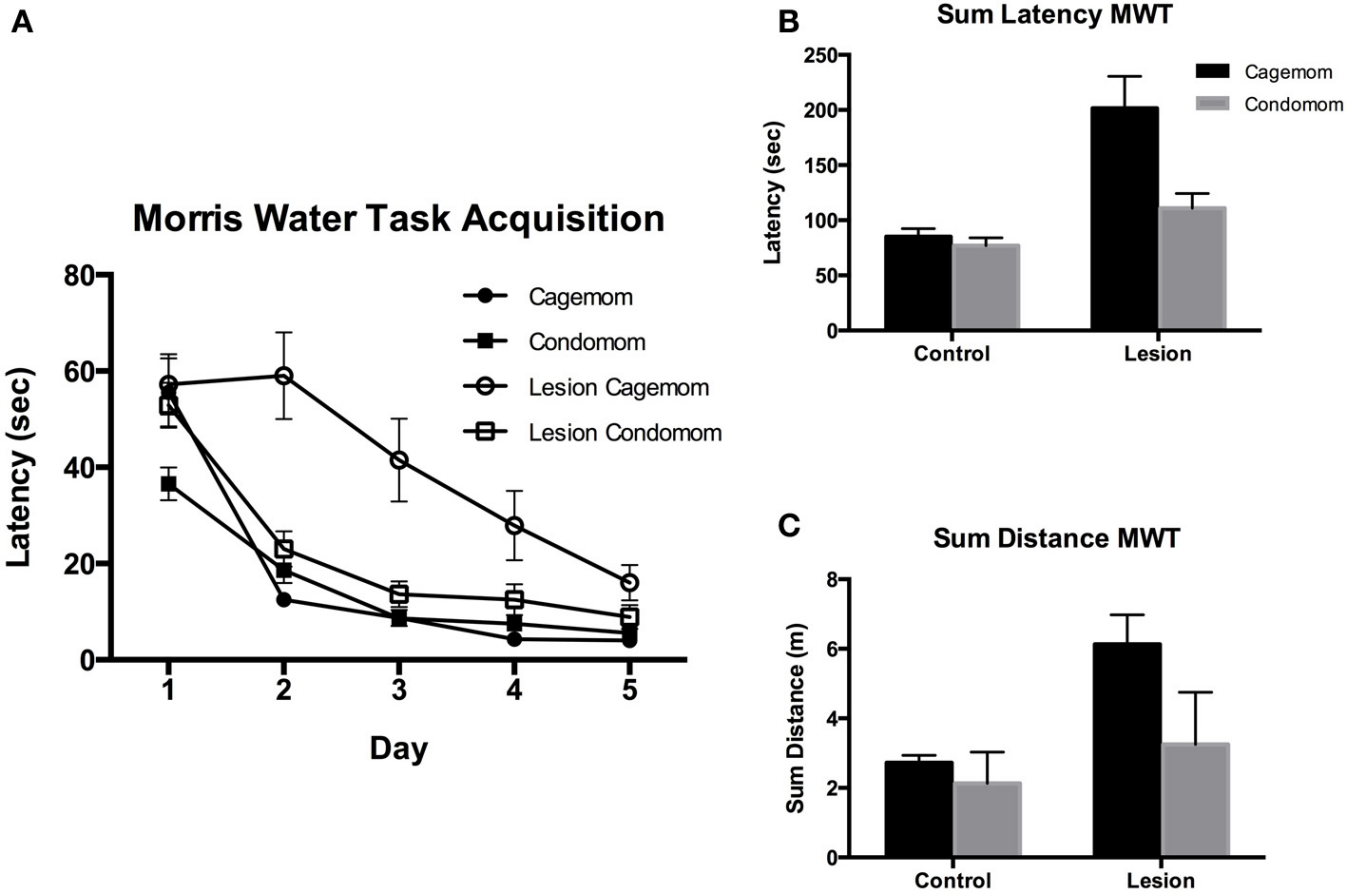
**(A)** Acquisition of learning the location of a hidden platform in the Morris Water. Task over 5 days of testing. Condomom animals with early lesions acquired the task faster than did cagemom animals with similar injury and did not differ from the performance of the cagemom controls. Latency is reported in seconds. (D, Days; C, Control; L, P3 Lesion). **(B)** Latency in seconds to find a hidden platform averaged and summed over 5 days of testing. Control animals found the platform faster than did animals with early lesions. Condomom animals with P3 lesions were significantly faster at finding the hidden platform than were P3 lesion animals without treatment. **(C)** Total distance swam (in arbitrary computer units) searching for a hidden platform in the Morris water task. Control animals swam shorter distances to find the platform and condomom lesion animals did not differ in swim distance from cagemom controls but swam shorter distances than their untreated counterparts.

***Latency***. Latency to find the hidden platform in this task has been shown to be an effective measure of cognitive impairment in P3 frontal cortex lesion animals (Kolb and Gibb, 2010). Consistent with the literature, in the current experiment it was found that animals with lesions of this type required more time to find the hidden platform than did their littermate controls. Notably, behavioral performance was enhanced in both the P3 lesion and the control animals that had prenatal complex housing experience (see Figures 2A,B). A Two-Way ANOVA revealed a main effect of group (lesion or sham) [*F*_(1, 61)_ = 25.3, *p* < 0.0001], treatment [*F*_(1, 61)_ = 8.0, *p* < 0.005] and a significant interaction [*F*_(1, 61)_ = 5.3; *p* < 0.05]. This result reflected the impairment shown by the P3 lesion animals at finding the hidden platform in the water maze and the effectiveness of prenatal complex housing in reversing that impairment. Further, *post-hoc* analysis revealed that although the condomom P3 lesion animals were still relatively impaired when compared to their control (sham-operates) cohorts (*p* = 0.03), they showed no impairment relative to the cagemom control group (*p* = 0.33) and were significantly better than the cagemom lesion animals (*p* = 0.0009).

***Distance***. The beneficial effect of prenatal experience was also manifested in the distance that the rats swam to find the platform (see Figure 2C). The untreated P3 lesion animals had the longest swim distances; the P3 condomom animals swam much less; and the control animals swam the least. A Two-Way ANOVA revealed a significant effect of group [*F*_(1, 61)_ = 22.5, *p* < 0.0001] and treatment [*F*_(1, 61)_ = 13.3, *p* = 0.0008], as well as the Group X Treatment interaction [*F*_(1, 61)_ = 5.8, *p* = 0.02]. The interaction reflected the observation that prenatal complex housing improved the performance of the lesion animals much more than the effect seen on the control animals. *Post-hoc* analysis of the data revealed that the distance the P3 condomom animals swam was significantly less than the distance swum by the cagemom P3 animals (*p* < 0.0001) and the P3 condomom animals did not differ from the cagemom controls (*p* = 0.46).

#### Whishaw tray reaching

**Figure 3:**
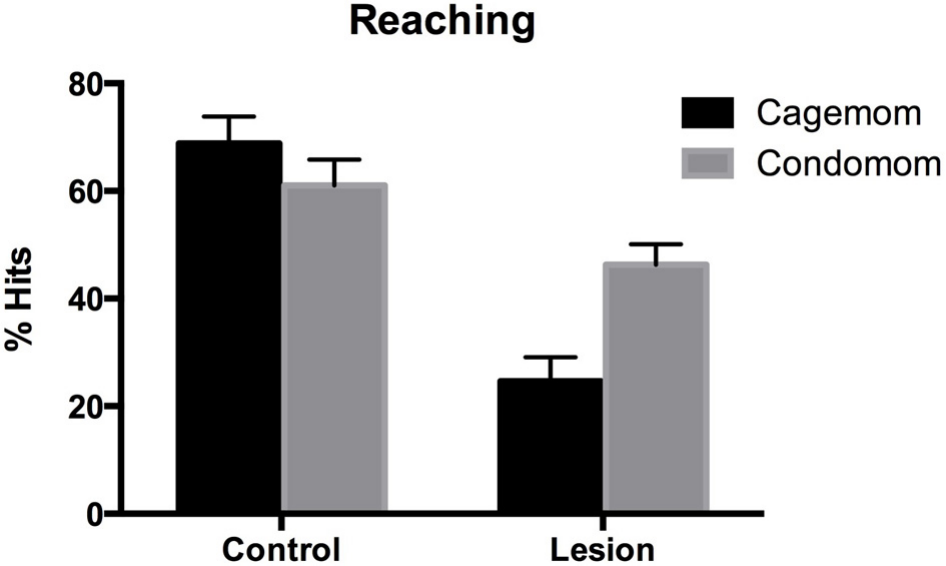
**Reaching success defined as the number of successful reaches divided by the total number of reaches (expressed as a percentage)**. Early frontal lesion disrupted reaching accuracy but condomom animals showed substantial recovery (or sparing) of reaching performance.

Two lesion-treated animals and one from the no treatment-lesion group refused to reach so they were excluded from the analysis. These three animals subsequently died and were not included in the anatomical analyses. Overall, lesion animals showed impairments in retrieving food pellets for consumption compared to littermate controls. Importantly, this impairment was reduced by prenatal complex housing. A Two-Way ANOVA with group and treatment as factors, showed that there was a main effect of group [*F*_(1, 58)_ = 39.8, *p* < 0.0001], a near-significant effect of treatment [*F*_(1,58)_ = 3.4, *p* = 0.06], and a significant interaction [*F*_(1, 58)_ = 6.02, *p* < 0.02]. This interaction reflected the observation that although prenatal complex housing did not improve the performance of control subjects, P3 lesion animals did benefit from the treatment (see Figure 3). A *post-hoc* analysis indicated that prenatal complex housing significantly improved performance of lesion animals (*p* = 0.016).

### Anatomical results

#### Body weight

Statistical analysis of adult body weight using a Three-Way ANOVA (group X sex X treatment) revealed that sex was the factor that had the greatest influence on body weight [*F*_(1, 58)_ = 49.6, *p* < 0.0001] (Table 1). The lesion animals showed reduced body weight [*F*_(1, 58)_ = 4.24, *p* = 0.044] when compared to controls but treatment showed no main effect on body weight [*F*_(1, 58)_ = 1.67, *p* = 0.20]. The only interaction in this analysis that was significant was the Group X Sex X Treatment interaction [*F*_(1, 58)_ = 3.93, *p* = 0.05]. This result reflected the trend for the condomom control males to be heavier than the cagemom control males and the condomom-lesion female animals to show a similar increase in body weight. In both cases the difference was in the order of 40–50 g.

**Table 1:** **Summary of body weights**.

**Group**	**Experience**	
	**Prenatal condo**	**No treatment**
Con. males	414.2 ± 21.1	365.3 ± 8.7
P3 males	332.4 ± 33.5	357.8 ± 22.4
Con. females	281.4 ± 8.6	274.6 ± 18.0
P3 females	287.0 ± 4.6	247.4 ± 11.4

Numbers refer to means ± standard errors in grams.

#### Brain weight

Exposure to complex housing during the prenatal period of development had no effect on brain weight in adulthood. A Three-Way ANOVA with lesion, sex, and treatment as factors revealed a main effect of lesion [*F*_(1, 58)_ = 84.4, *p* < 0.0001] wherein the lesion animals had a reduced brain weight compared to controls and a main effect of sex [*F*_(1, 58)_ = 8.26, *p* = 0.0056] as male rats had larger brains than their female counterparts (Table 2). This result reflects the sexual dimorphism normally seen in brain weight. There was no main effect of treatment [*F*_(1, 58)_ = 1.79, *p* = 0.187], however, nor were any of the interactions significant (*p*'s > 0.3)

**Table 2:** **Summary of brain weight**.

**Group**	**Experience**	
	**Prenatal condo**	**No treatment**
Con. males	2.009 ± 0.027	1.958 ± 0.007
P3 males	1.752 ± 0.034	1.743 ± 0.054
Con. females	1.909 ± 0.025	1.900 ± 0.064
P3 females	1.716 ± 0.017	1.652 ± 0.053

Numbers refer to means ± standard errors in grams.

#### Cortical thickness

To determine regional effects of lesion and treatment the analysis of cortical thickness was divided into two groups. The first group included anterior planes 1 and 2, and the second group included posterior planes 3–5. The P3 frontal lesion reduced cortical thickness across all planes measured whereas cortical thickness was increased in the anterior sections by condomom treatment.

A Two-Way ANOVA on the anterior planes revealed a significant main effect of group [*F*_(1, 58)_ = 110.0, *p* < 0.0001] and treatment [*F*_(1, 58)_ = 18.7, *p* < 0.0001]. The interaction of Group X Treatment was also significant [*F*_(1, 58)_ = 7.4, *p* = 0.008]. The interaction reflected the finding that the lesion caused less reduction in cortical thickness in the condomom animals than in the cagemom animals (Table 3).

**Table 3:** **Summary of cortical thickness at anterior and posterior planes**.

**Planes**	**Group**	**Experience**	
		**Prenatal condo**	**No treatment**
1–2	Con	42.2 ± 0.5	41.3 ± 0.4
	P3	38.0 ± 0.6^*^	34.2 ± 0.5
3–5	Con	34.1 ± 0.3	34.1 ± 0.4
	P3	31.5 ± 0.2	31.4 ± 0.4

Numbers refer means ± standard errors in mm.

* Differs significantly from no treatment values in same lesion group (p < 0.05).

A Two-Way ANOVA on the posterior planes revealed a significant effect of group [*F*_(1, 58)_ = 64.2, *p* < 0.0001] but not treatment [*F*_(1, 58)_ = 0.06, *p* = 0.81] nor the interaction [*F*_(1, 58)_ = 0.095, *p* = 0.76].

#### Lesion size

**Figure 4:**
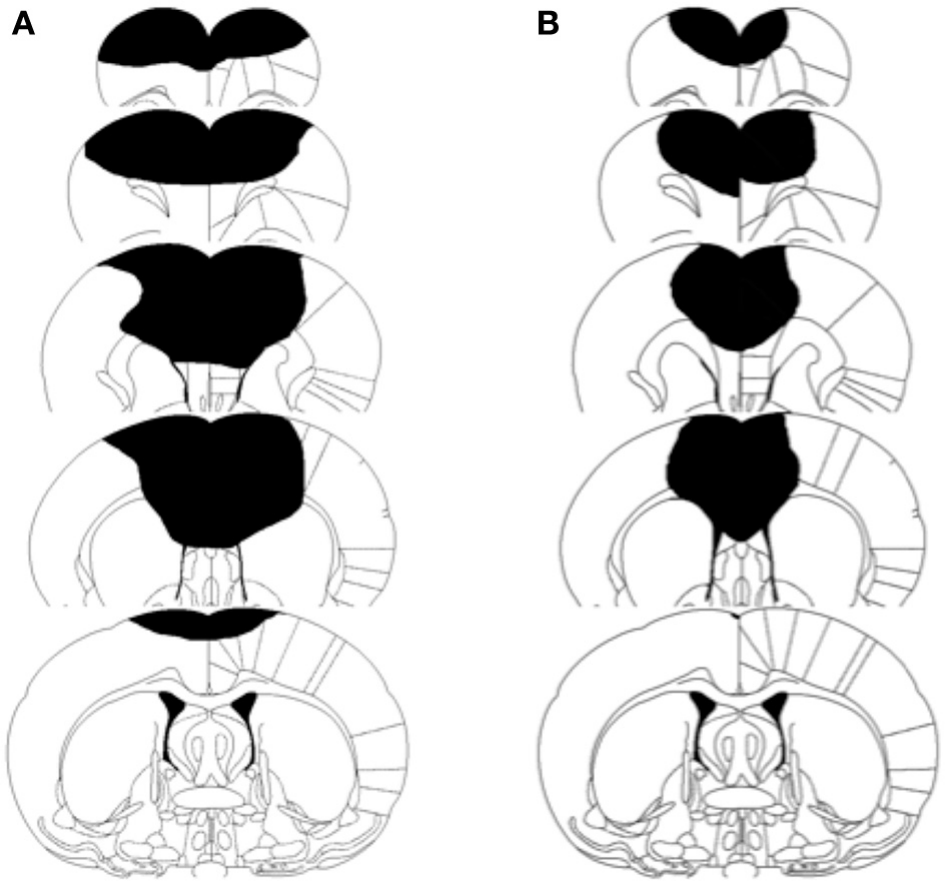
**Schematic illustration of lesion size in two representative brain injured animals**. **(A)** Represents cagemom animal. **(B)** Represents condomom animal.

A One-Way ANOVA revealed that the prenatal complex housed P3 operates had significantly smaller lesions than did the untreated P3 operates [*F*_(1, 35)_ = 6.72, *p* = 0.014]. The condomom animals had lesions that averaged 8.0% of the cerebral hemispheres whereas the cagemom animals had lesions that averaged 17.8% of the cerebral hemispheres (see Figure 4). This difference in lesion size is not likely due to variations in the size of lesion made at the time of surgery. The surgeon (B.K.) has more than 30 years experience in making this type of lesion and was blind to the treatment conditions of the animals at the time of the surgery.

#### Thalamic measures

Only animals that had a clear thalamic representation on both planes outlined in the methods section were included in the analysis. The P3 lesion caused a reduction in thalamic area in the cagemom animals. Both the control and lesion groups in the prenatal condo-housed condition showed a larger thalamic compared to the untreated animals. At the anterior plane of measure, a Two-Way ANOVA showed a significant main effect of group [*F*_(1, 47)_ = 5.6, *p* = 0.022] and treatment [*F*_(1, 47)_ = 22.1, *p* < 0.0001] but no interaction [*F*_(1, 47)_ = 3.05, *p* = 0.087]. There was an increase of 9% in the control animals and of 19.5% in the lesion animals following prenatal maternal enrichment. Although the interaction was not significant there was an obvious trend for the P3 lesion to reduce thalamic area less in the condomom group than in the cagemom group (Table 4).

**Table 4:** **Summary of thalamic area in the anterior plane**.

**Plane**	**Experience**	
	**Prenatal condo**	**No treatment**
Control	108.9	100.0
P3 lesion	107.1	87.6^*^

Numbers refer to percent of no treatment control values.

* Differs significantly from no treatment control values (p < 0.05).

A Two-Way ANOVA on the thalamic measures derived from the posterior plane showed no significant effect of group [*F*_(1, 47)_ = 2.07, *p* = 0.16] but the effect of treatment was highly significant [*F*_(1, 47)_ = 23.0, *p* < 0.0001]. Prenatal maternal enrichment increased thalamic size by 16.2% in the control animals and 20.4% in the lesion animals over their non-enriched cohorts. The interaction of these factors was not significant [*F*_(1, 47)_ = 0.33, *p* = 0.57]. As was observed in the anterior plane, the prenatally complex housed animal showed larger thalamic areas than did the untreated animals (Table 5).

**Table 5:** **Summary of thalamic area in the posterior plane**.

**Plane**	**Experience**	
	**Prenatal condo**	**No treatment**
Control	116.2^*^	100.0
P3 lesion	112.8^*^	92.4

Numbers refer to percent of no treatment control values.

* Differs significantly from no treatment control values (p < 0.05).

## Golgi-cox analysis

### Spines

#### Apical dendrites

Analysis of spine density on the apical tree using a Two-Way ANOVA with lesion and treatment as factors revealed a significant effect of lesion [*F*_(1, 58)_ = 4.26, *p* = 0.043] and treatment [*F*_(1, 58)_ = 6.69, *p* = 0.012]. The interaction of Lesion X Treatment was non-significant [*F*_(1, 58)_ = 0.95, *p* = 0.33]. The P3 lesion caused an increase in spine density in the operate animals. The effect of treatment was to increase spine density as well, so the animals that were prenatally condo-housed had the largest increase in spine density (Table 6). The largest effect of treatment was seen in the lesion animals however a trend in the same direction was noted in the control animals (*p* = 0.02).

**Table 6:** **Summary of spine density on the apical terminal**.

**Group**	**Experience**	
	**Prenatal condo**	**No treatment**
Control	6.32 ± 0.24	5.95 ± 0.20
P3 lesion	7.02 ± 0.17^*^	6.20 ± 0.30

Numbers refer to number of spines/10 μm dendritic branch length.

* Differs significantly from no treatment control values (p < 0.05).

#### Basilar dendrites

A Three-Way ANOVA with group, treatment, and sex as factors revealed that there was no group effect [*F*_(1, 54)_ = 0.035, *p* = 0.853] on spine density on basilar dendrites but there was a main effect of sex [*F*_(1, 54)_ = 6.954] and of treatment [*F*_(1, 54)_ = 40.69, *p* < 0.0001]. None of the interactions reached the level of significance. Overall, females had a higher spine density than males. Males and females exposed to prenatal complex housing in both control and lesion conditions had more spines than did untreated animals (Table 7).

**Table 7:** **Summary of spine density on the basilar terminal**.

**Group**	**Experience**	
	**Prenatal condo**	**No treatment**
Control-male	7.31 ± 0.24^*^	5.77 ± 0.19
Control-female	7.58 ± 0.21^*^	6.62 ± 0.12
P3 lesion-male	7.29 ± 0.41^*^	5.94 ± 0.16
P3 lesion-female	7.69 ± 0.45^*^	6.50 ± 0.49

Numbers refer to number of spines/10 μm dendritic branch length.

* Differs significantly from no treatment control values of the same sex (p < 0.05).

## Discussion

Numerous studies have shown the benefits of postnatal enrichment on both brain and behavior (for a review see Johnson et al., 2013). The present study is the first to demonstrate the powerful prophylactic effect that *prenatal* enrichment has on early cortical injury. Animals with P3 frontal lesions born to mothers that experienced complex housing during pregnancy displayed remarkable recovery in both cognitive and motor domains compared to their non-treated cohorts. Associated with this behavioral recovery, condomom offspring showed decreased lesion size, increased thalamic area, increased cortical thickness, and increased spine density. These results are discussed in the following sections.

### Prenatal experience alters behavior in control animals and recovery after cortical injury

Prenatal complex housing significantly improved the behavioral performance of P3 frontal cortex lesion animals. Recovery was to the extent that the lesion animals did not differ from controls in the Morris water task. In fact, this treatment also enhanced cognitive performance in animals that did not sustain a lesion. These findings suggest that prenatal complex housing influenced mechanisms underlying brain plasticity that translated into behavioral improvements in both control and lesion animals. Previous studies have demonstrated the robust nature of the Morris water task in assessing spatial cognition (Morris, 1981; D'Hooge and De Deyn, 2001) and detecting behavioral deficits in animals with neocortical and hippocampal damage (Sutherland et al., 1989; Kolb and Gibb, 1991; Gonzalez et al., 2002). In our developmental investigations we have shown that early lesions to the frontal, cingulate, or parietal cortices result in consistent deficits in Morris water task acquisition (Gonzalez et al., 2003; Gibb and Kolb, 2005; Kolb and Gibb, 2010). These studies have demonstrated that such deficits can be ameliorated by other experiential treatments, such as tactile stimulation or postnatal enrichment (Comeau et al., 2008; Gibb et al., 2010). However, the extent to which animals in the current study recovered, and the benefit that non-lesion animals experienced, strongly suggest that prenatal enrichment is remarkably effective for enhancing cognitive performance.

Animals were also assessed for motor function using the Whishaw tray-reaching task (Whishaw et al., 1986, 1991). This task measures the rat's ability to use their forepaws to retrieve food. A rat is trained to reach through metal bars to retrieve chicken feed from a tray at the front of the cage. This test is specific for motor skill and performance is measured by the success of the animal to retrieve and ultimately consume food. Several studies have shown that control animals performance asymptote around 60% accuracy (Gonzalez and Kolb, 2003; Gharbawie et al., 2005; Gibb et al., 2010). It is possible that because the animals in the current study were at this level further enhancement of this behavior may not be possible due to a ceiling effect. Numerous investigations have shown that animals with cortical injury to the frontal or parietal regions are impaired in the task (Gonzalez and Kolb, 2003; Whishaw et al., 2004; Gharbawie et al., 2005). This is particularly true for animals that sustain early perinatal (P0–P5) frontal cortex injury (Kolb and Cioe, 2000; Kolb et al., 2000; Gibb and Kolb, 2005). Supporting previous findings, our lesion animals that received no treatment showed abysmal performance on the task, displaying a success rate of about 25%. In contrast, condomom lesion animals displayed a success rate that almost doubled that of non-treated animals. Behavioral improvement in this task has been reported in animals with similar cortical injury after tactile stimulation of the pups post-injury (Kolb and Gibb, 2010). What is notable is that animals in the current investigation did not receive any direct remediation *per se*.

Together, the cognitive and motor improvement seen in the condomom offspring strongly suggests that the complex-housing of the mothers acted as a prophylactic for the pups that received frontal cortex lesions shortly after birth. What is not known is whether the enhanced behavioral performance of the animals resulted from a reduced lesion effect or a potentiated recovery.

### Prenatal experience affects brain organization: underpinnings of behavioral improvement?

Although there is considerable evidence that prenatal experiences alter brain development, this evidence is primarily related to the effects of maternal stress or chemical exposure. For example, exposing pregnant animals to stress significantly changes brain function and structure in offspring (Mychasiuk et al., 2011a,b; Weinstock, 2011; for a review see Weinstock, 2011). With respect to prenatal chemical exposure, alcohol for example, has well-known teratological effects on brain development (Goodlett et al., 2005; Kelly et al., 2009). A single injection of bromodeoxyuridine at E12-E15 produces widespread changes in cerebral structure and function (Kolb et al., 2012). Prescription drug exposure during pregnancy in humans and other animals is also well known to affect anatomical and functional brain outcomes in offspring (Andrade, 2013a,b; Ungerer et al., 2013; Liew et al., 2014). The unique finding of the current study is the demonstration that sensory-motor experience of a dam during her pregnancy can influence the brain development and function in her offspring throughout the lifespan. More significant is the fact that this influence can be *beneficial* to both control and brain-injured offspring.

Prenatal complex housing exerted marked anatomical brain changes on both control and lesion offspring. Control animals showed 9% increase in thalamic area at the anterior plane and a 16.2% increase at the posterior plane. This result demonstrates the potent effect of prenatal experience on brain plasticity. Condomom lesion animals showed smaller lesions, increased thalamic area, attenuated reduction of cortical thickness, and increased spine density in both apical and basilar trees. The finding that lesion size was considerably smaller in prenatal complex housed animals suggests that there may have been a reduction in apoptotic cell death near the lesion site. Alternatively, the prenatal complex housing may have changed the expression of growth factors in the brain and as a result stimulated postnatal neuronal proliferation to partly compensate for the tissue loss. We have shown previously that rats with medial frontal lesions around days 7–10 do show spontaneous neurogenesis (e.g., Kolb et al., 1998) so it is possible that the prenatal treatment has somehow made the brain respond more like a brain with a lesion in the second week of life, but this remains conjecture at this point.

Remarkably, thalamic area increased at both anterior and particularly posterior planes in lesion condomom animals. The reason for the increased thalamic area is unclear but it could be related to changes in thalamocortical connectivity. Higashi et al. (2002) found thalamocortical synaptic connections to be functional by E19 in rats and the authors suggested that these prenatal thalamocortical connections could influence cortical circuitry before birth. So it is possible that maternal enrichment altered patterns of thalamic connectivity. The nature of the changes and their functional significance after brain injury is only a matter of speculation at this point. But Hayward et al. (2010) have shown increased vessel density and blood flow in thalamus in animals that show functional recovery after traumatic brain injury. It is possible that similar changes occur after P3 cortical lesions, particularly after prenatal enrichment. Furthermore, increased thalamic area might be partly responsible for the smaller lesion cavity, the thicker cortex and the increased spine density observed in the prenatally enriched lesion animals.

In the current study and as reported previously, frontal cortex lesions on P3 led to a dramatic reduction in cortical thickness (Gibb and Kolb, 2005; Kolb and Gibb, 2010). Prenatal complex housing however, mitigated this reduction significantly. Complex housing after weaning has also been shown to reduce the effects of P3 lesions on cortical thickness (Comeau et al., 2008). One can speculate that enhanced neural connectivity in the prenatally enriched animals may at least in part account for this finding. In fact this speculation is also supported by the increase in spine density observed in the parietal cortex of these animals, particularly in the basilar dendritic field. In this case, there was a dramatic increase in spine density as a result of the prenatal enrichment in both control and lesion animals. Remarkably, mother's enrichment experience increased spine density in the basilar tree of all the animals with the highest increase seen in the male controls. In this case, the increase in spine density represents approximately a 26% increase in excitatory synapses. The Golgi results suggest that the intrinsic cortical organization may be fundamentally different in the animals born to prenatally complex housed mothers. Further studies that look at neuronal number, size, and complexity, are necessary to confirm this speculation.

In sum, *prenatal* enrichment increased thalamic area, cortical thickness, and spine density in parietal cortex. These dramatic changes are likely responsible for at least some of the observed behavioral improvements seen in control and lesion animals. In a recent study we have shown epigenetic changes in offspring associated with maternal prenatal enrichment. Specifically there were significant reductions in global DNA methylation in the hippocampus and frontal cortex. This reduction in DNA methylation implies an increase in gene expression across enrichment groups and sexes. It is tempting to speculate that methylation changes in the hippocampus produce changes in gene expression that support neurogenesis (Kempermann et al., 2002). This may in turn contribute to behavioral improvement in the Morris Water task (Arai and Feig, 2011). It can also be hypothesized that the DNA methylation changes in the frontal cortex are at least in part responsible for the recovery observed in the lesion animals on skilled motor behavior (Nygren and Wieloch, 2005).

Although a potential limitation of the current study is the lack of cross-fostering of the pups, this procedure has traditionally been used in studies where the prenatal maternal experience is negative (e.g., exposure to drugs or stress). Because the current was an enrichment experiment, we had no reason to believe that maternal care would be negatively impacted. In addition, studies using cross-fostering show that this method does not always mitigate the effects of prenatal experience on development of the offspring (e.g., Bauco and Rompre, 2006; Howells et al., 2009; Clinton et al., 2010; Schroeder and Weller, 2010; Slamberova et al., 2010, 2011). Finally, it has been shown that cross-fostering can serve as an enriching experience (van der Veen et al., 2008). Further experiments are warranted to address these issues.

In conclusion, we have shown in a long series of studies that perinatal cortical injury produces a consistent array of anatomical effects including a decrease in brain size, decreased cortical thickness, reduced dendritic arborization, reduced spine density, and abnormalities in cortical connectivity (e.g., Kolb, 1995). These anatomical changes can be influenced, and partly reversed, by postnatal experiences such as complex housing (e.g., Kolb, 1995). Furthermore, the various anatomical changes are correlated with a severe behavioral syndrome that also is partly ameliorated by the postnatal experiences. The novel finding of the current studies is that prenatal experience can alter the way the brain responds to later injury. This finding has important implications for the prenatal management of infants expected to be a risk for difficult birth.

### Conflict of interest statement

The authors declare that the research was conducted in the absence of any commercial or financial relationships that could be construed as a potential conflict of interest.
